# Male Partner's Involvement in HIV Counselling and Testing and Associated Factors among Partners of Pregnant Women in Gondar Town, Northwest Ethiopia

**DOI:** 10.1155/2016/3073908

**Published:** 2016-07-31

**Authors:** Alemu Zenebe, Abebaw Gebeyehu, Lemma Derseh, Kedir Y. Ahmed

**Affiliations:** ^1^Department of Pediatric Operation Theatre, Black Lion teaching Hospital, Addis Ababa, Ethiopia; ^2^Department of Reproductive Health, Institute of Public Health, College of Medicine and Health Science, University of Gondar, P.O. Box 196, Gondar, Ethiopia; ^3^Department of Epidemiology and Biostatistics, Institute of Public Health, College of Medicine and Health Science, University of Gondar, P.O. Box 196, Gondar, Ethiopia; ^4^Department of Public Health, College of Medicine and Health Science, Debre Markos University, P.O. Box 269, Debre Markos, Ethiopia

## Abstract

*Background.* Despite the existence of several programmes promoting male involvement in HIV counselling and testing during their wife's pregnancy as a part of PMTCT, few men have heeded the call. The aim of this study was to assess male partner's involvement in HCT and its associated factors.* Methods*. This study was based on institution based cross-sectional study design that used systematic random sampling technique. A total of 416 partners were interviewed in the data collection. Multivariable logistic regression model was fitted to identify the independent predictors.* Result*. In this study, the prevalence of male involvement in HCT was found to be 40.1% (95% CI: 35.3%–44.7%). The independent predictors of male involvement were partners who were younger, were cohabitant, were with multigravida wives, were knowledgeable on route of mother-to-child transmission, and discussed HCT.* Conclusion*. The prevalence of male involvement in HCT was found to be suboptimal compared to similar studies in Ethiopia. There is a need of interventions on partners who are older, separated, and with lower gravidity wife. Awareness creation campaign should also be created on the route of mother-to-child transmission of HIV and on the importance of discussion with wife.

## 1. Introduction

HIV pandemic created an enormous challenge to the survival of mankind worldwide [1]. Worldwide there are an estimated 33.3 million people infected with HIV; Sub-Saharan Africa bears the greater burden with an estimated 22.5 million people infected with HIV [2]. According to UNAIDS, women represent 52% of those infected with HIV worldwide and in Sub-Saharan Africa 60% of those infected with HIV are women [2]. With a national adult HIV prevalence of 1.5% (1.9% in women and 1.0% in men), Ethiopia is one of the country's most severely hit by the epidemic [3].

Mother-to-child transmission (MTCT) is an important source of HIV infection among Ethiopian children, which accounts for more than 90% of pediatric AIDS [1, 4]. Prevention of Mother-to-Child Transmission (PMTCT) programmes have been proven to be effective in reducing the risk of HIV transmission from infected mothers to their children [5]. Without intervention, the risk of MTCT of HIV ranges from 20% to 45%. With specific interventions in nonbreastfeeding populations, the risk of MTCT can be reduced to less than 2% and to 5% or less in breastfeeding populations [6]. Antenatal care (ANC) is a major entry point for PMTCT programmes especially in countries with a high prevalence of HIV. It creates an opportunity to capture pregnant mothers and their male partners to reverse the transmission of HIV during pregnancy, labour, and breastfeeding [7]. Male involvement is necessary for improving women's uptake of core PMTCT services; it is a key contributor to community acceptance and support of PMTCT [8].

However, actual involvement of male partners in PMTCT programmes in several counties of Sub-Saharan Africa is low and programmes report difficulties in attracting the involvement of male partners. Studies conducted in African countries showed that male involvement in PMTCT during pregnancy ranges from 11% to 58.3% [5, 9–11]. In Ethiopia, studies conducted in Arba Minch and Debre Markos town reported that 53.6% and 55.4% of partners accompanied their wives for HIV testing and counselling [12, 13].

Despite the existence of several programmes promoting male involvement in HIV counselling and testing during their wives' pregnancy as a part of PMTCT, few men have heeded the call. Numerous reasons for nonparticipation of male partners in HIV counselling and testing as PMTCT programmes have been gained from studies that primarily focused on women as respondents. This study primarily focused on men to gain understanding of factors that influence their involvement in these programmes. Thus, this study was aimed at assessing male involvement and associated factors in HCT among partners of pregnant women in Gondar town, Northwest Ethiopia.

## 2. Methods

### 2.1. Study Design and Study Setting

Institution based cross-sectional study was used to assess the prevalence and associated factors of male involvement in HCT among partners of pregnant women in Gondar administrative city from July to November 2014. Gondar is an ancient town located 727 Km North of Addis Ababa with an estimated population of 207,044 of which 50.8% were females and the rest are males. There are eight health centers that provide ANC service with HCT of both pregnant women and their husbands.

### 2.2. Source and Study Population

Partners of all pregnant women who did voluntary HCT in the current pregnancy were the study population. Those partners selected by systematic random sampling technique were included in this study. Partners who are unable to communicate for different reasons and those who are not living together were excluded from the study.

### 2.3. Sample Size Determination and Sampling Procedure

By using single population proportion formula, as there is no information about it, about 50% prevalence of male involvement was used. Considering 5% margin of error and 95% confidence interval with 10% nonresponse rate, the final minimum sample size was found to be 422. Based on the number of mothers who were on ANC follow-up, the samples were distributed to all eight health centers proportionate to their number. After the first participant was selected using lottery method, systematic random sampling technique was used to select eligible participants.

### 2.4. Data Collection

Data was collected by using pretested structured questionnaire. The tool was developed in English and translated to Amharic and then back to English to check for its consistency. Ten data collectors and three supervisors who can speak Amharic language were recruited. Partners involved in HCT were interviewed at the facility level and those who did not involve in HCT were at the household level. To assure the quality of the data, three-day training was given to data collectors and supervisors, and on each data collection day some percent of the collected data were reviewed by principal investigator; any problems faced in the time of data collection were discussed and immediate solution was made.

### 2.5. Operational Definitions

Operational definitions are as follows:

- Male involvement: when husbands or partners of pregnant women attended both HIV counselling and testing (HCT) during ANC visit for the purpose of PMTCT service.
- Knowledge about route of MTCT: when respondents know at least one route of transmission of MTCT of HIV from three questions.
- Knowledge about PMTCT: when respondents know at least one way of PMTCT from three questions.

### 2.6. Data Processing and Analysis

The data were checked, coded, and entered to Epi-info software version 3.5.1 and cleaning was performed by using SPSS 20. Frequencies, proportions, and measures of central tendency and variation were used to describe the study participant. Binary logistic regression was used to examine association between dependent and each independent variable. All variables with *p* < 0.2 in bivariate analysis were entered into multiple logistic regression model to identify factors independently associated with male involvement in HCT during pregnancy. Backward stepwise likelihood ratio was used to select the final independent predictors. The significance of Odds Ratios (OR) was determined with 95% CI and *p* < 0.05.

### 2.7. Ethical Consideration

Ethical clearance was obtained from University of Gondar, and letter of permission was obtained from the respected health institutions. Informed consent from each study participant was being obtained after explaining the purpose of the study. Confidentiality of the information was assured by omitting names from the questionnaire and maximum effort was made to maintain privacy of the respondents during the interview. No question was asked about their serostatus. No incentives were provided to the respondents as a way of motivating them to participate in the study. All the identified potential participants agreed to participate in the study.

## 3. Result

### 3.1. Sociodemographic Characteristics of Partners

The nonresponse rate of this study was found to be 6 (1.42%). The mean (±SD) age of partners who participated in this study was 34.38 years (±6.0). The majority, 210 (50.5%) of them, reported the current pregnancy as wife's first pregnancy (Table 1).

Among respondents, 206 (49.5%) reported that the average distance of the living area from health institution used to perform HCT was less than five Km. Regarding the amount of money they paid for transports, 182 (43.8%) of the them paid less than five Ethiopian birr for the trip (Table 1).

### 3.2. Knowledge and Attitude towards PMTCT

About 386 (92.8%) of them knew at least one route of MTCT and 301 (78%), 298 (77.2%), and 199 (51.6%) of them reported MTCT of HIV during pregnancy, breastfeeding, and childbirth, respectively. The majority, 384 (92.3%) of respondents, knew at least one method of PMTCT and 324 (84.4%) of them knew that provision of ARTs for the mother could help to reduce MTCT of HIV. On the other hand, 221 (57.6%) of them knew that avoiding breastfeeding is one of the alternatives for preventing HIV transmission from mother to child, but only 3.4% [12] of respondents were aware that risk of MTCT of HIV could be reduced by caesarean section. Among respondents, 375 (90.1%) of them knew the presence of HCT for pregnant women during their ANC visit. Three hundred ninety-six (95.2%) of them agreed on the necessity of partner testing. About 356 (85.6%) of partners knew that discordant result can be found among married partners (Table 2).

### 3.3. Perceived Risk to Acquire the Virus

Perceived risk of acquiring the virus was reported in about 98 (23.6%) of male partners. Of these, 57 (58.2%) used unsterile sharp object and 44 (44.9%) of them had multiple sexual partners (Table 2).

### 3.4. Level of Male Involvement in HCT

Among partners, 278 (66.8%) discussed HCT with their pregnant wives. And more than two-thirds (69.2%) of them had willingness to accompany PMTCT clinic with their pregnant wife together. Two hundred twelve (51%) of them visited PMTCT clinic with their wife. About 174 (41.8%) of respondents were involved in the counselling part only and 167 (40.1%) of them with 95% CI (35.2–44.2) participated in both counselling and testing, meaning the overall involvement in HCT (Table 3). Among those involved in HCT, 100 (59.9%) were involved because they felt responsibility. Work overload which was mentioned by 131 (52.6%) of them was the main reason for noninvolvement of partners (Table 3).

### 3.5. Factors Associated with Male Involvement in HCT

In multivariable analysis, being at younger age group, couples living together, wife's number of pregnancies, having knowledge on the timing of MTCT, and husbands discussing HCT with their wives were positively and significantly associated with male involvement in HCT. The odds of male involvement in HCT was higher in 20–29-year (AOR = 4.94 95% CI: 1.97–12.39) and 30–39 years' (AOR = 2.82 95% CI: 1.37–5.81) age groups as compared to those who were 40 years of age and above.

Those partners who are living together with their wives were 5.5 (95% CI: 1.97–15.39) times more likely to be involved in HCT. Wife's gravidity of 2-3 (AOR = 5.34 95% CI: 1.38-20.69) and 4-5 (AOR = 8.10 95% CI: 1.52-43.32) was significantly associated with male involvement in HCT compared to those who had only one pregnancy. The likelihood of male involvement in HCT was found to be higher (AOR = 7.41 95% CI: 1.80–30.45) among those who knew the timing of mother-to-child transmission. Those partners who reported discussion about HCT with their wives had increased (AOR = 8.60 95% CI: 4.30–17.21) odds of involvement in HCT compared to their counterparts (Table 4).

## 4. Discussion

In this study, about 40.1% of the partners escorted their wives to ANC and received HIV counselling and testing together. This finding is relatively similar to study conducted in Addis Ababa and Tanzania community which was 44% and 46.3%, respectively [9, 14]. However, the finding is lower than that reported in Cameroon which was 58.3% [5], while it was higher than pooled estimate of studies conducted in India, Cameroon, Georgia, and the Dominican Republic which was 36.1% and another study conducted in Cape Town, South Africa, which was 32% [11, 15]. Thus, the finding of this study implies that there is already an encouraging platform for male involvement in the study area, and this could serve as a springboard to achieve full scale male involvement in HCT in the city of Gondar and other similar urban areas.

This study demonstrated that the level of male involvement in HCT was found to be higher among younger male partners. This finding is consistent with reviewed literatures which found that males involved in HCT were younger than those who received HCT alone in the same clinic [16]. This might be due to increased communication between couples and level of knowledge expected to be reduced with age. This is supported by study conducted in South Africa, 23.5% of individuals 50 years of age and above did not know the route of transmission of HIV from mother to child [17]. Moreover, an operational research conducted in Zimbabwe showed that as age increased majority of men fear going for HIV tests [18]. However, similar studies conducted in Cameroon and Western Uganda showed that the proportion of males accompanying their partner increased with age, for example, in rural western Uganda males older than 35 years were 2.89 times more likely to receive VCT than those of 35 years or younger [5, 19]. The difference with these studies could be explained by the existence of social support and difference in health service utilization in the later studies.

This result showed that male partners of pregnant women who were living with their wives were significantly more likely to be tested than those partners of pregnant women living in separated place. Absence of the male partner for discussion at home and decreased likelihood of accompanying his pregnant wife during ANC follow-up could be the possible explanation for the above finding. In this study, male involvement in HCT was significantly associated with wife's number of pregnancies. This could be explained by the fact that for each additional pregnancy there is increased frequency of contact of mothers with health care workers which increases their awareness and their chance of discussion with their husbands. Furthermore, these findings contradict with studies conducted in India and Kenya and showed that males who had fewer children were more likely to assist their partner in pregnancy and childbirth than males who had large number of children [20, 21].

Those partners who know at least one mode of transmission of HIV from MTC were 7.4 times more involved in HCT compared to their counterparts. This finding is similar to the study conducted in Zambia which showed that knowledge and the total score on level of involvement were positively and significantly associated [22]. This might be due to increased level of knowledge and awareness about HCT expected to have a positive influence on men's involvement in HCT. Having history of discussion about HIV testing with pregnant wife remained significantly associated with male attendance at the antenatal clinic for HCT. This finding was similar to another study conducted in Zambia [22]. Having discussion with their wives might help them to get what they heard from the health care workers during their wives' ANC visit which could be the possible explanation for increased uptake of partners.

The possibility of social desirability bias due to sensitiveness of issues and cross-sectional nature of the study which fails to show causal relationship were among the limitations of this study. Study was conducted both in health institution and in community level to address both involvers and noninvolvers which is the strength of this study.

## 5. Conclusion

Despite the existence of several programmes promoting male involvement in HIV counselling and testing during their wives' pregnancy as a part of PMTCT, still lower proportion of them accompany their wives for HCT. The prevalence of male involvement was found to be significantly higher among partners who are younger, living with their wives, are living with multigravida wives, are knowledgeable about mode of mother-to-child transmission of HIV, and discussed HCT with their wives. Therefore, there is a need of an intervention in the independent predictors.

## Figures and Tables

**Table 1:** A sociodemographic characteristic of partners of pregnant women in Gondar town, Northwest Ethiopia, 2013.

Variables	Frequency	Percentage (%)
Age		
20–29	79	19
30–39	237	57
40+	100	24
Marital statues		
Married	403	96.9%
Unmarried	13	3.1%
Religion		
Orthodox christian	287	69
Muslim	97	23.3
Others (protestant or catholic)	32	7.7
Educational status		
No formal education	66	15.9
Primary education	97	23.3
Secondary and above	253	60.8
Occupation		
Private business	143	34.4
Government employee	88	21.2
NGO	74	17.8
Daily labor	62	14.9
Farmer and others	49	11.8
Wife's number of pregnancies		
1	166	39.9
2-3	210	50.5
4-5	34	8.2
6+	6	1.4
Time of living together		
Not living together	11	2.6
1–5 years	254	61.1
6–10 years	108	26
11–15 years	33	7.9
16+ years	10	2.4
Distance to nearby health facility		
Less than 5 km	206	49.5
5–10 km	128	30.8
More than 10 km	82	19.7
Money paid for transportation		
Did not pay	34	8.2
<5 Ethiopian birr	182	43.8
5–10 Ethiopian birr	165	39.6
>10 Ethiopian birr	35	8.4

**Table 2:** Knowledge and attitude towards PMTCT and risk perception of male partners of pregnant women in Gondar town, Northwest Ethiopia, 2013.

Variables	Frequency	Percentage (%)
Occurrence of MTCT (*n* = 386)		
During pregnancy	301	78
During childbirth	199	51.6
During breastfeeding	298	77.7
PMTCT methods (*n* = 384)		
ART	324	84.4
Caesarean section	13	3.4
Avoiding of breastfeeding	221	57.6
Presence of HCT during ANC visit		
Yes	375	90.1
No	16	3.9
I do not know	25	6
Necessity of partner testing		
Yes	396	95.2
No	20	4.8
Discordant test result		
Yes	356	85.6
No	60	14.4
Feeling when seen with pregnant women in clinic		
Feel nothing	214	51.4
Feel happiness	197	47.4
Feel ashamed	5	1.2
Risk perception		
Yes	98	23.6
No	318	76.4
Reasons of risk perception (*n* = 98)		
Used unsterile sharp object	57	58.2
Had multiple sexual partner	44	44.9
Had sexual contact without condom	39	39.8
Had sex with positive person	10	10.2

**Table 3:** Level of involvement of partners of pregnant women towards HCT in Gondar town, Northwest Ethiopia, 2013.

Variables	Frequency	Percentage (%)
Discussed about HCT with their wife		
Yes	278	66.8
No	138	33.2
Willingness to visit PMTCT clinic with his wife		
Yes	288	69.2
No	128	30.8
Visited PMTCT clinic with his wife		
Yes	212	51
No	204	49
Involved in counselling only		
Yes	174	41.8
No	242	58.2
Involved for both counselling and testing		
Yes	167	40.1
No	249	59.9
Reasons for partners involvement (*n* = 167)		
Feel responsibility	100	59.9
Initiated by provider	65	38.9
Initiated by wife	44	26.3
Reasons for partners noninvolvement (*n* = 249)		
Work overload	131	52.6
Fear of acquiring the virus	116	46.6
Confidentiality issue	47	18.9
Fear of inaccurate test result	12	4.8

**Table 4:** Factors associated with male involvement in HCT in Gondar town, Northwest Ethiopia, 2013.

Variables	Male involvement		COR with 95% CI	AOR with 95% CI
	Yes	No		
Age				
20–29	36	43	2.51 (1.33, 4.73)	**4.94 **(1.97,12.39)^**∗∗**^
30–39	106	131	2.43 (1.44, 4.08)	**2.82 **(1.37,5.81)^**∗**^
40+	25	75	1	1
Living together				
Yes	161	199	6.74 (2.82, 16.12)	**5.50 **(1.97,15.39)^**∗∗**^
No	6	50	1	1
Wife's total pregnancy				
One	66	100	1	1
Two-three	87	123	1.07 (0.71, 1.62)	**5.34 **(1.38,20.69)^**∗**^
Four-five	13	21	0.94 (0.44, 2.0)	**8.10 **(1.52,43.32)^**∗**^
Six and above	1	5	0.30 (0.04, 2.65 )	1.35 (0.09, 20.23)
Knew time of MTCT				
Yes	164	222	6.65 (1.98, 22.29)	**7.41 **(1.80,30.45)^**∗**^
No	3	27	1	1
Discussed HCT				
Yes	153	125	10.84 (5.94, 19.77)	**8.60 **(4.30,17.21)^**∗**^
No	14	124	1	**1**

^*∗*^ *p* value < 0.05, ^*∗∗*^ *p* value <= 0.001, COR = crude odds ratio, and AOR = adjusted odds ratio.
